# Deep Learning–Derived Retinal Vascular Morphology and Incident Treatment for Sight-Threatening Diabetic Retinopathy

**DOI:** 10.1016/j.xops.2026.101385

**Published:** 2026-08-26

**Authors:** Paul Nderitu, Mark Chia, Urvi Karamchandani, Christine A. Kiire, Asterios Diafas, Sarah Matta, Yukun Zhou, Pearse A. Keane

**Affiliations:** 1 Moorfields Eye Hospital NHS Foundation Trust, London, United Kingdom; 2 NIHR Biomedical Research Centre, Moorfields Eye Hospital NHS Foundation Trust, London, United Kingdom; 3 Institute of Ophthalmology, University College London, London, United Kingdom; 4 Department of Ophthalmology, Royal Victorian Eye & Ear Hospital, Canberra, Australia; 5 Oxford University Hospitals NHS Foundation Trust, Oxford Eye Hospital, Oxford, United Kingdom; 6 Université de Bretagne Occidentale, Brest, France; 7 The UCL Hawkes Institute, University College London, London, United Kingdom; 8 Department of Computer Science, University College London, London, United Kingdom

**Keywords:** Retinal vascular morphology, Diabetic retinopathy, AutoMorph, Deep learning, Prognostic biomarkers

## Abstract

**Objective:**

Retinal vascular morphology (RVM) features could be useful biomarkers for risk stratification for incident sight-threatening diabetic retinopathy (STDR). We evaluated whether deep learning–derived macula-centered RVM, extracted at scale, are associated with incident treatment for STDR including proliferative diabetic retinopathy (PDR) and clinically significant macula edema (CSME).

**Design:**

Retrospective longitudinal cohort study (2000–2022).

**Participants:**

We included 20 855 patients (33 861 eyes) with gradable, treatment-naïve eyes with a macula-centered fundus image at baseline.

**Methods:**

Arterial and venous RVM features of complexity, density, caliber, and tortuosity were extracted using an automated deep learning tool (AutoMorph). Multivariable Cox proportional hazards models were performed to quantify the association between RVM and incident treatment for STDR, adjusting for age, sex, ethnicity, baseline diabetic retinopathy (DR) and diabetic macula edema (DME) grade, and intereye correlation using a cluster term.

**Main Outcome Measures:**

Incident treatment for STDR including PDR or CSME.

**Results:**

Over a mean 1.74 (standard deviation 2.15) years of follow-up, 3446 eyes (10.2%) received treatment for STDR (1387 PDR; 2059 CSME). Higher arterial complexity and density were strongly protective for incident STDR treatment (arterial complexity Q4 vs. Q1 hazard ratio [HR] 0.52 [95% confidence interval 0.45–0.60]; arterial density HR 0.47 [0.41–0.54]). Larger venous caliber was associated with an increased risk of incident STDR treatment (HR 2.53 [2.23–2.86]), while increasing arterial caliber was associated with a reduced risk of incident PDR treatment (HR 0.64 [0.53–0.77]). There were weak associations between increasing arterial and venous tortuosity and an increased risk of incident treatment for PDR and CSME, respectively (arterial tortuosity HR 1.11 [95% confidence interval 1.01–1.22]; venous tortuosity HR 1.16 [1.03–1.31]). Retinal vascular morphology feature associations were generally stronger for incident treatment for PDR than CSME. Associations between RVM and incident treatment for STDR remained consistent after a sensitivity analysis which only included patients with no or mild DR and no DME at baseline.

**Conclusions:**

Macula-centered RVM features show clinically plausible associations with incident treatment for STDR. Retinal vascular morphology features extracted using an automated deep learning tool are potential prognostic biomarkers that could be useful for DR risk prediction models alongside other retinal imaging biomarkers and clinical risk factors.

**Financial Disclosure(s):**

Proprietary or commercial disclosure may be found in the Footnotes and Disclosures at the end of this article.

Sight-threatening diabetic retinopathy (STDR), comprising proliferative diabetic retinopathy (PDR) and clinically significant macula edema (CSME), drives the majority of vision loss in patients with diabetes.^1^^,^^2^ Therefore, the early identification of eyes at high risk of progression to STDR could enable targeted surveillance and timely treatment, in turn improving visual outcomes and efficiently allocating health care resources. A growing body of evidence suggests that retinal vascular morphology (RVM) features, including vessel complexity, caliber, tortuosity, and branching are potential biomarkers for diabetic retinopathy (DR) development and progression.^3^, ^4^, ^5^, ^6^, ^7^, ^8^, ^9^, ^10^, ^11^, ^12^, ^13^, ^14^, ^15^, ^16^, ^17^, ^18^ Studies have shown changes in RVM are associated with other diabetic complications including neuropathy,^19^^,^^20^ nephropathy,^21^ and cerebrovascular disease.^22^ However, the translation of RVM features into clinical practice has been constrained by 3 key challenges: (1) imaged fundus region: many prior studies used optic disc–centered fundus images focusing on peripapillary metrics including the central retinal artery equivalent and central retinal vein equivalent, whereas routine diabetic eye care relies on macula-centered fundus images; (2) sample size and precision: most prior studies included <1000 participants resulting in wide confidence intervals or nonsignificant effect estimates; and (3) semiautomated RVM feature extraction: prior studies used more labor intensive computer-assisted RVM feature extraction methods that require input from human graders.^3^, ^4^, ^5^, ^6^, ^7^, ^8^, ^9^, ^10^, ^11^, ^12^, ^13^, ^14^, ^15^, ^16^, ^17^, ^18^

Over the last 2 decades, RVM extraction analysis tools have been developed including Singapore I Vessel Assessment, QUantitative Analysis of Retinal vessel Topology and size, and Vessel Assessment and Measurement Platform for Images of the REtina, among others, demonstrating the widespread interest in RVM.^9^^,^^18^^,^^22^, ^23^, ^24^ Building on these foundations, AutoMorph, an open-access deep learning tool for reliable automated artery/vein RVM feature extraction was developed.^25^ By combining open accessibility, scalability, and robust validation, AutoMorph enables the large-scale assessment of RVM associations with a diverse set of disease processes in clinical data sets.^26^, ^27^, ^28^, ^29^

The Moorfields Diabetic imAge dataSet Study data set, which contains longitudinal retinal imaging and ophthalmic clinical data from 37 279 patients with DR between 2000 and 2022, provides a unique opportunity to test the hypothesis that macula-centered, deep learning–derived RVM features are associated with incident STDR. Unlike prior studies, we explicitly contrast our approach along the axes of RVM extraction method (automated deep learning tool), scale (n ≈ 20 k vs. ∼1 k patients, a x20 increase), and imaged fundus region (macula-centered vs. optic disc–centered).^3^, ^4^, ^5^, ^6^, ^7^, ^8^, ^9^, ^10^, ^11^, ^12^, ^13^, ^14^, ^15^, ^16^, ^17^, ^18^ Precise estimates of the association between RVM features with STDR could help identify retinal microvascular biomarkers of DR progression to inform the future development of reliable risk prediction models to personalize DR care. The primary objective of this study is to evaluate the association between macula RVM features and incident treatment for STDR including treatment for PDR and CSME.

## Methods

### Study Design, Population, and Data

We performed a retrospective, longitudinal cohort study using the Moorfields Diabetic imAge dataSet Study data set (January 2000–March 2022). The study included adult patients with diabetes graded for DR or receiving intravitreal treatment for CSME, who underwent routine retinal imaging as part of standard care at Moorfields Eye Hospital NHS Foundation Trust (Fig 1). Demographic information (age, sex, and ethnicity), DR grade, diabetic macula edema (DME) grade, and macula-centered fundus images were extracted (Fig 1). At baseline, eligible eyes had DR and DME grades, were naïve for PDR/CSME or treatment for STDR, and had a gradable macula-centered color fundus image acquired with a Topcon 3-dimensional OCT fundus device. For participants with multiple eligible macula images, the first gradable macula image was selected for analysis. Diabetic retinopathy grading was performed by multidisciplinary clinicians (retinal specialists and optometrists) as part of routine clinical care using the International Clinical Diabetic Retinopathy Disease Severity Scale. The Automorph tool, which automatically detects image gradability, was also used to exclude ungradable images which were consistently classified as “reject” by an ensemble of grading models (Fig 1). For eligible gradable images, AutoMorph segmented arteries and veins, then produced quantitative metrics for RVM features of artery and vein complexity (fractal dimension), density (vessel density), caliber (average vessel width), and tortuosity (distance tortuosity) (Fig 1).^25^

**Figure 1:**
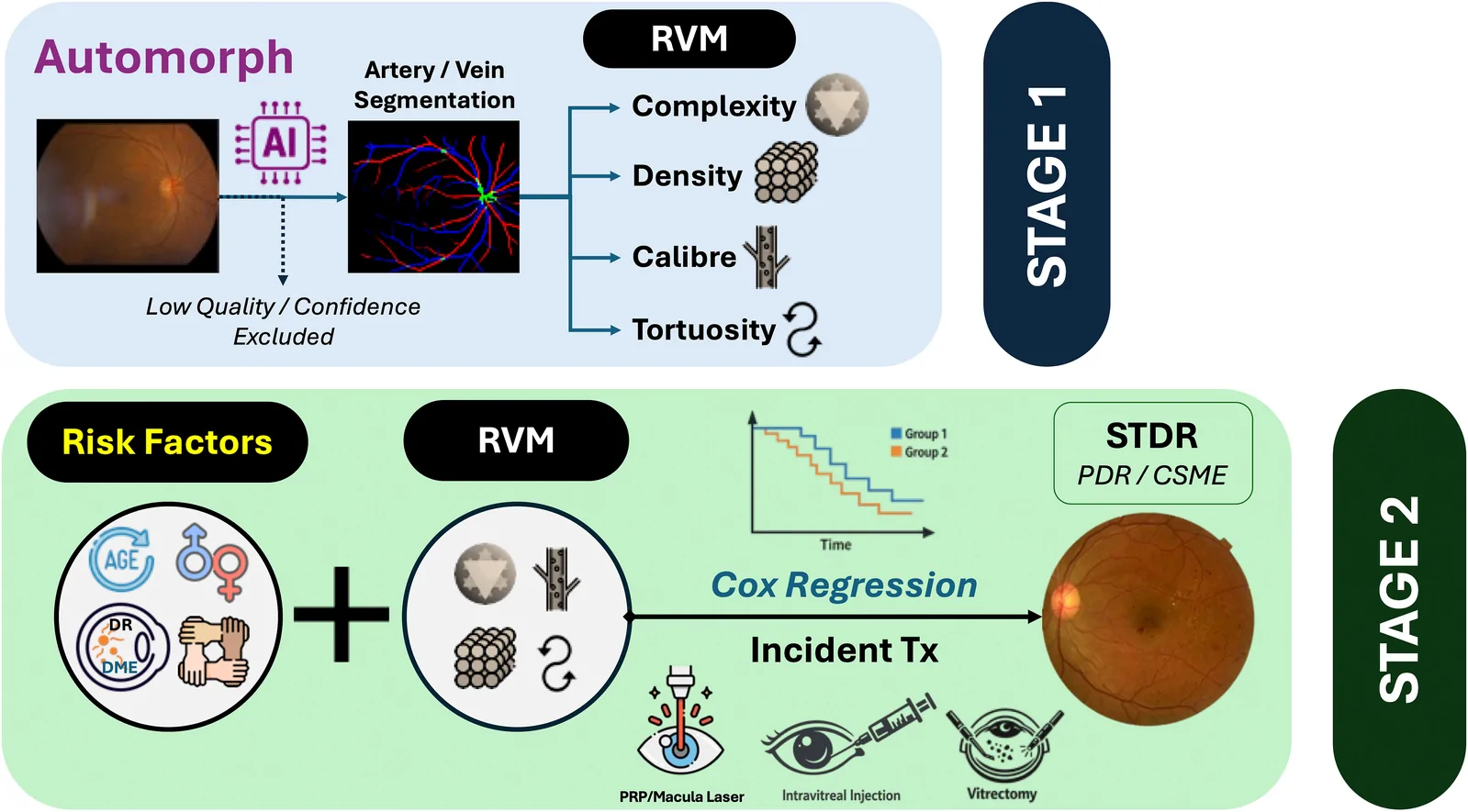
Study overview stage 1: Automated extraction of retinal vascular morphology (RVM) is performed by the AutoMorph deep learning tool with the exclusion of low-quality images. For gradable macula-centered fundus images, artery and vein segmentation is performed then extraction of RVM metrics of complexity (fractal dimension), density (vessel density), caliber (average vessel width), and tortuosity (distance tortuosity). Stage 2: The association between deep learning–derived RVM features with incident treatment (Tx) for sight-threatening diabetic retinopathy (STDR) including proliferative diabetic retinopathy (PDR) or clinically significant macular edema (CSME) is assessed using a multivariate Cox regression analysis adjusting for baseline risk factors including age, sex, and baseline DR/DME grades. AI = artificial intelligence; DME = diabetic macula edema; DR = diabetic retinopathy; PRP = panretinal photocoagulation.

### Outcomes

The primary study outcome was incident treatment for STDR, defined as the first occurrence of treatment for either PDR (pan retinal photocoagulation, intravitreal anti-VEGF injection with PDR-related procedures (e.g. pan retinal photocoagulation), vitrectomy, or delamination surgery) or CSME (intravitreal anti-VEGF/steroid implants/steroid injections without PDR-related procedures, or focal/grid macula laser) (Fig 1).

### Statistical Analysis

Baseline characteristics were summarized using means and standard deviations for continuous variables and counts (%) for categorical variables. There were no missing values for age or sex, while missing values for ethnicity were categorized as “not specified” and cases included. Correlations were computed between RVM features and age, baseline DR grade, and DME grade using Pearson correlation coefficient. Multivariate Cox proportional hazards models estimated associations between baseline RVM quartiles and time to incident treatment for STDR, adjusting for age, sex, ethnicity, baseline DR and DME grade, and an eye cluster term. The time origin was defined from the baseline eligible macula image. Patients were censored at the date of their final recorded follow-up visit or at the study's hard endpoint (March 2022) if no treatment event occurred. Intereye correlation was handled using a cluster term for eyes from the same patient. Proportional hazards assumptions were verified using Schoenfeld residuals which were visually inspected. Adjustment for baseline DR and DME grades removed the effect of baseline retinal ischemia or edema allowing for the evaluation of the independent effect of RVMs. The RVM features were modeled in separate analyses due to the high correlation between respective RVM features, but both respective arterial and venous RVM were included in the same model to quantify vessel-specific contributions. Finally, a sensitivity analysis was performed to assess the association between RVM quartiles and time to incident treatment for STDR for patients with only no or mild DR and no DME at baseline adjusting for age, sex, ethnicity, baseline DR (mild), and an eye cluster term. Prespecified subgroup analyses for PDR and CSME outcomes were also performed. Statistical significance was determined using a 2-sided alpha of 0.05. Analyses were conducted in R (R Foundation for Statistical Computing) and Python (Python Software Foundation).

## Results

### Baseline Characteristics

From 37 279 patients (73 746 unique eyes), 25 867 patients (69%; 45 652 eyes) with ≥1 gradable macula-centered fundus image and valid RVM metrics were initially selected. We excluded 11 412 patients (31%; 28 094 eyes) with ungradable images or those not from a Topcon device. After excluding patients without a valid baseline DR or DME grade, the study cohort comprised 20 855 patients (56%) and 33 861 treatment/STDR-naïve eyes (Fig 2). The mean (standard deviation) age was 61.3 (14.2) years; 42.7% female with and ethnicity distribution of White 14.4%; Black 8.5%; South Asian 21.7%; mixed 0.9%; other 25.2%. At baseline, DR status was 25.1% no DR, 53.7% mild nonproliferative DR (NPDR), 15.2% moderate NPDR, and 6.0% severe NPDR. Mild DME was present in 13.1% of eyes (Table 1). Baseline RVM feature distributions showed vessel complexity, density, and caliber were significantly greater for retinal veins than arteries, but arterial tortuosity was significantly greater than venous tortuosity (Fig S1, available at www.ophthalmologyscience.org). There were strong positive correlations observed between corresponding arterial and venous complexity and density, and respective vessel calibers (Fig S2, available at www.ophthalmologyscience.org). Increasing age was moderately negatively correlated with both arterial and venous complexity and density. A sample of macula images from the lowest (Q1) and highest (Q4) RVM feature quartile for arteries and veins are shown in Figure S3 (available at www.ophthalmologyscience.org).

**Figure 2:**
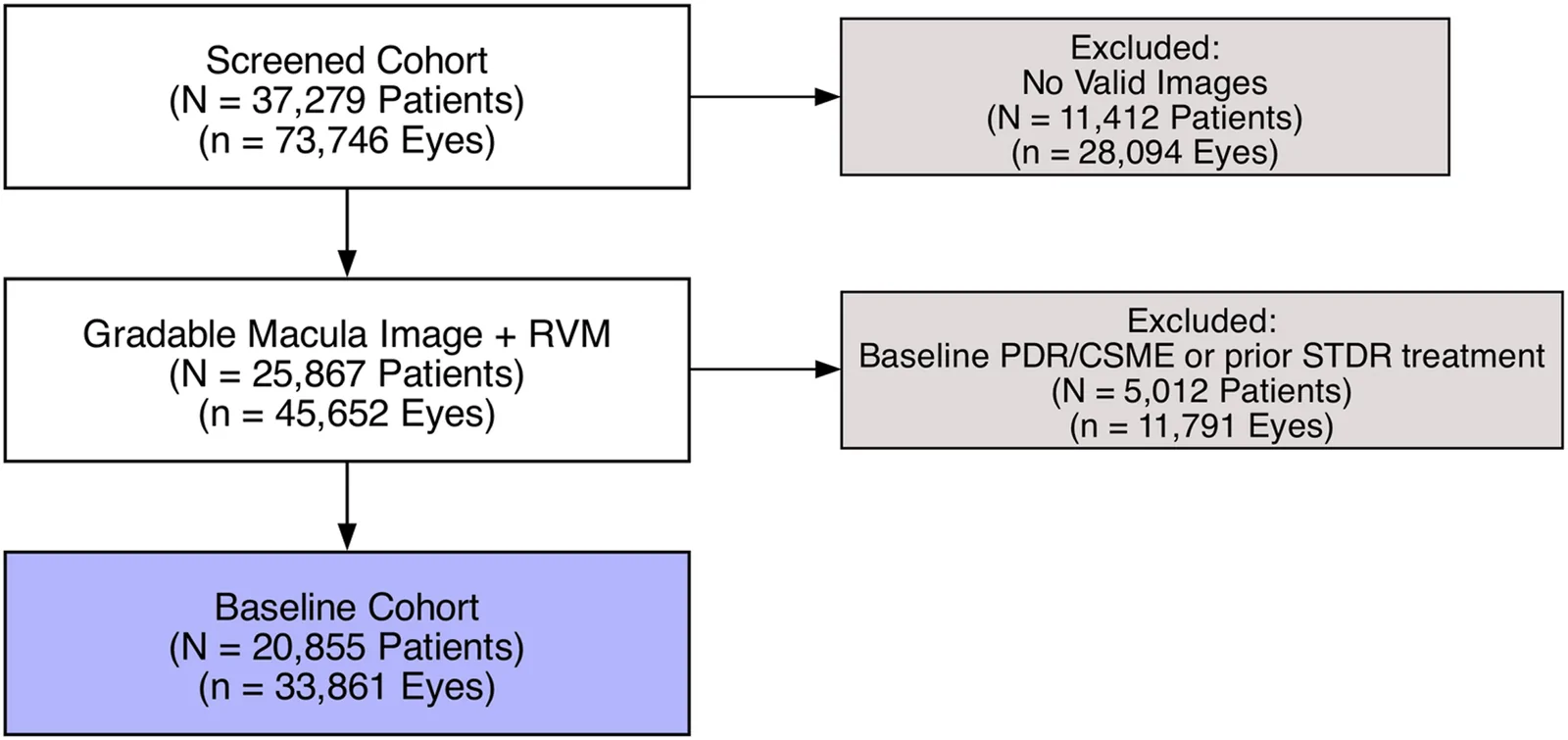
Study flowchart. CSME = clinically significant macula edema; PDR = proliferative diabetic retinopathy; RVM = retinal vascular morphology; STDR = sight-threatening diabetic retinopathy.

**Table 1:** Baseline Cohort Characteristics

Characteristic	Mean (SD) or N [%]
Age (yrs)	61.27 (14.16)
Sex	
Female	14 459 [42.7%]
Male	19 402 [53.7%]
Ethnicity	
White	4880 [14.4%]
Black	2869 [8.5%]
South Asian	7337 [21.7%]
Mixed	306 [0.9%]
Other	8526 [25.2%]
Not specified	9943 [29.4%]
DR grade	
No DR	8488 [25.1%]
Mild NPDR	18 189 [53.7%]
Moderate NPDR	5148 [15.2%]
Severe NPDR	2036 [6.0%]
DME grade	
No DME	29 426 [86.9%]
Mild DME	4435 [13.1%]
Follow-up duration (yrs)	1.74 (2.15)

DME = diabetic macula edema; DR = diabetic retinopathy; NPDR = nonproliferative diabetic retinopathy; SD = standard deviation.

### Associations between RVM Features and Incident Treatment for STDR

Over a mean 1.74 (standard deviation 2.15) years of follow-up, 3446 eyes (10.2%) received treatment for STDR, comprising 1387 PDR treatments and 2059 CSME treatments.

#### Complexity

Higher vessel complexity (artery and vein) was strongly associated with a lower risk of incident treatment for STDR (Fig 3 and Table S1, available at www.ophthalmologyscience.org). Compared with the lowest quartile (Q1), vessel complexity hazard ratios (HR) [95% confidence interval] for incident treatment for STDR were arterial Q4 HR 0.52 [0.45–0.60] and venous Q4 HR 0.66 [0.57–0.75]. Associations were consistent when analyzed separately for incident treatment for PDR and CSME, with the strongest effect observed for arterial complexity and incident treatment for PDR; Q4 HR 0.44 [0.36–0.55]. Sensitivity analysis did not show changes in associations between artery or vein complexity and incident treatment for STDR in patients with no or mild baseline DR (Table S2, available at www.ophthalmologyscience.org).

**Figure 3:**
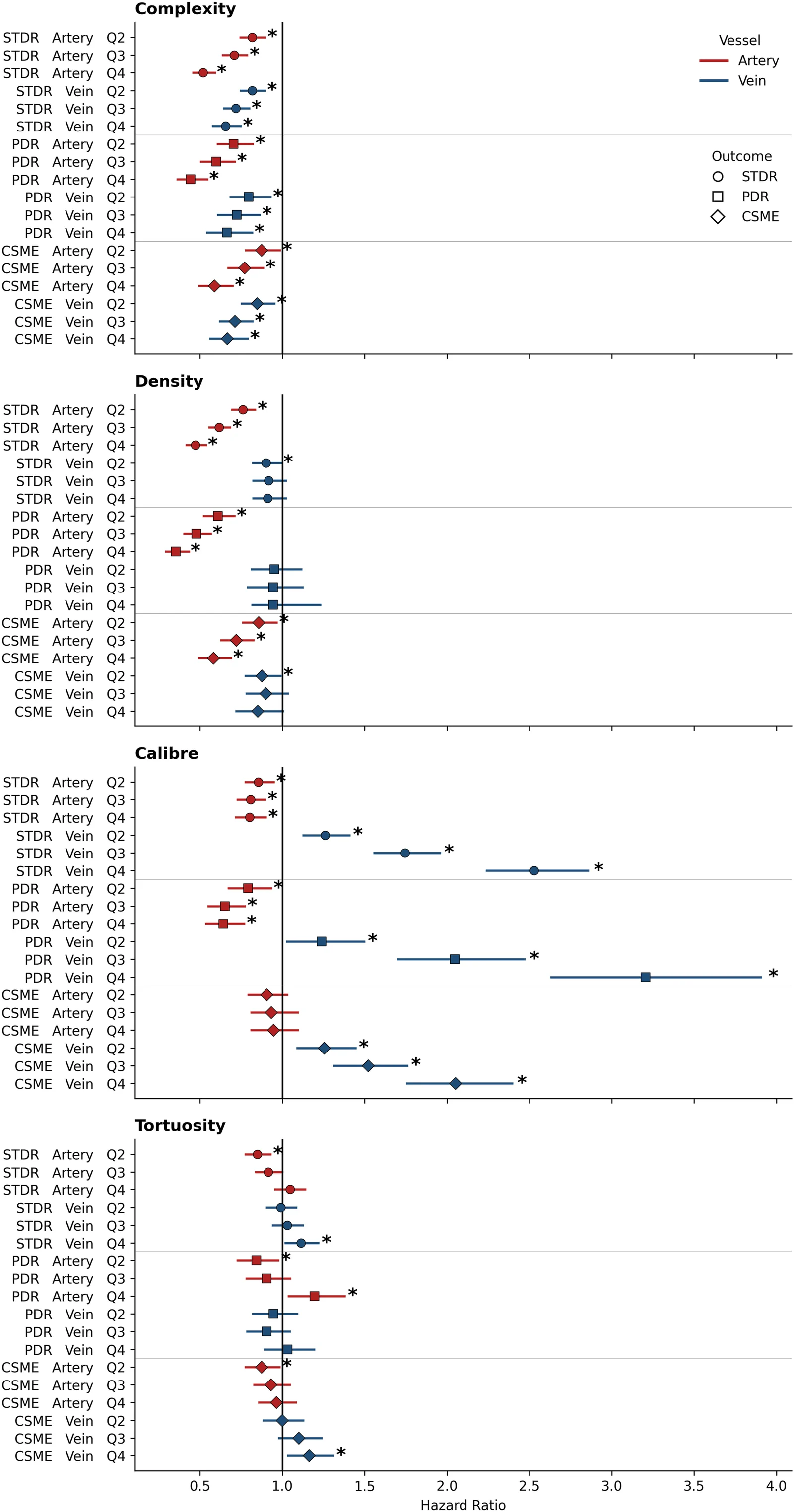
Retinal vessel morphology features and incident treatment for STDR hazard ratio forest plot showing association between RVM features and incident treatment for STDR. Increasing vessel complexity (artery and vein) associated with a reduced risk of STDR treatment. Increasing artery density associated with a reduced risk of STDR treatment. Higher artery caliber associated with a reduced risk of STDR treatment but, conversely, higher vein caliber is associated with an increased risk of STDR treatment. Increasing vessel tortuosity (artery and vein) only weakly associated with an increased risk of PDR and CSME treatment, respectively. complexity: fractal dimension, density: vessel density, caliber: average vessel width, tortuosity: distance tortuosity, Q: Quartile. Quartile 1 is the reference group. Multivariate Cox proportional hazards model adjusted for age, sex, ethnicity, baseline DR grade, baseline DME, and a cluster term to adjust for intereye correlation. Significant results (*P* < 0.05) are indicated with a star (∗). CSME = clinically significant macula edema; DME = diabetic macula edema; DR = diabetic retinopathy; PDR = proliferative diabetic retinopathy; RVM = retinal vessel morphology; STDR = sight-threatening diabetic retinopathy.

#### Density

Higher arterial density was associated with a lower risk of incident treatment for STDR (Fig 3 and Table S1); arterial Q4 HR 0.47 [0.41–0.54]. The association was strongest between arterial density and incident treatment for PDR Q4 HR 0.35 [0.29–0.44]. Less consistent associations were found between venous density and incident treatment for STDR. Significant associations between artery density and incident treatment for STDR remained in patients with baseline no/mild DR (Table S2).

#### Caliber

Vessel caliber demonstrated opposing associations with incident treatment for STDR between arteries and veins (Fig 3 and Table S1). Increasing arterial caliber reduced the risk of incident treatment for STDR; Q1 versus Q4 HR 0.80 [0.71–0.90], while increasing venous caliber increased risk; Q4 HR 2.53 [2.23–2.86]. The strongest effect was observed for venous caliber with incident treatment for PDR; Q4 HR 3.20 [2.63–3.91]. Vessel caliber associations with incident treatment for STDR showed similar directions in the sensitivity analysis compared to the main analysis (Table S2).

#### Tortuosity

Arterial and venous tortuosity showed weaker and less consistent associations with incident treatment for STDR (Fig 3 and Table S1). Increasing arterial tortuosity displayed a modest inverse association in Q2 HR 0.85 [0.77–0.93] with a trend toward higher risk in Q4 HR 1.05 [0.95–1.14], potentiated largely by associations with incident treatment for PDR; Q4 HR 1.19 [1.03–1.38]. Increasing venous tortuosity showed a small positive association; Q4 HR 1.11 [1.01–1.22], driven mainly by the association with incident treatment for CSME; Q4 HR 1.16 [1.03–1.31]. Associations remained inconsistent and weak between vessel tortuosity and incident treatment for STDR in the sensitivity analysis (Table S2).

## Discussion

In this large, longitudinal cohort we found that deep learning–derived macula RVM features were strongly associated with incident treatment for STDR, independent of the effect of demographics, baseline DR, and baseline DME grade. Specifically, higher vessel complexity and arterial density were independently associated with a reduced risk of incident treatment for STDR. Larger venous and smaller artery caliber were associated with a higher risk of incident STDR treatment. Greater vessel tortuosity showed weak associations with incident treatment for STDR. Associations remained consistent after a sensitivity analysis which included patients with no or mild DR and no DME to eliminate the effect of established retinal ischemia or macula edema.

Our robust estimates extend and strengthen previous evidence linking RVM with DR progression. While prior investigations were limited by small sample sizes (typically <1000 participants) and relied on manual or semiautomated vessel measurements from disc-centered fundus images, our study scaled the sample size by a factor of x20 and used more clinically relevant macula-centered fundus images from a diverse ethnic population.^3^, ^4^, ^5^, ^6^, ^7^, ^8^, ^9^, ^10^, ^11^, ^12^, ^13^, ^14^, ^15^, ^16^, ^17^, ^18^^,^^21^^,^^24^ We also validated the use of a fully automated deep learning tool, AutoMorph, for RVM feature extraction to this use case, in turn demonstrating its utility and scalability.^25^ Consistent and biologically plausible associations between arterial and venous RVM features were found with incident treatment for STDR, especially PDR treatment, supporting the hypothesis that retinal microvascular rarefaction and vessel simplification predispose to DR progression.

Prior population-based studies such as the Wisconsin Epidemiologic Study of Diabetic Retinopathy and the Australian Diabetes, Obesity and Lifestyle Study (AusDiab) demonstrated that larger retinal venular caliber and narrower arterioles were associated with a higher risk of incident and progressive DR, in line with our findings.^11^^,^^15^^,^^17^^,^^30^ Longitudinal data from other studies have also showed that progressive venular dilation preceded the onset of PDR, and is predictive of progression even after adjustment for glycemic control and diabetes duration.^12^^,^^13^^,^^16^^,^^31^^,^^32^ Studies have also reported that retinal vascular geometry, including complexity, tortuosity, and branching angles, offer additional prognostic value for DR progression. Lim et al^5^ (2017) reported that lower vessel complexity and wider venular branching angles were associated with incident DR, while Forster et al^4^ found that both increased venular tortuosity and reduced complexity predicted incident DR in adults with type 2 diabetes. Similar associations were observed in younger type 1 diabetes cohorts, where vascular remodeling during adolescence was predictive of subsequent retinopathy development.^12^^,^^14^^,^^17^^,^^31^ Collectively, these studies suggest that venular dilation, reduced vascular complexity, and retinal vascular geometric changes are markers of retinal microvascular compromise.

The inverse relationship between arterial complexity and vessel density with STDR treatment suggest that vessel rarefaction predicts the onset of severe DR. This may correlate with capillary dropout and vessel closure driven by chronic hyperglycaemia.^33^ Conversely, the strong positive association between venous caliber and STDR treatment risk aligns with hemodynamic alterations and endothelial dysfunction that accompany DR, consistent with the hypothesis that venular dilation represents a compensatory response to retinal hypoxia.^32^ Together, these patterns imply that both arterial rarefaction and venous dilation serve as complementary imaging biomarkers of DR progression. The associations between macula RVM features and STDR treatment were generally stronger for PDR than CSME. This suggests that vascular complexity and density changes are stronger predictors of proliferative disease than vascular permeability. Weaker associations observed for vascular tortuosity imply that it is a less specific biomarker of DR progression.

Fundus images mainly depict larger superficial retinal vessels in a 2-dimensional projection, whereas OCT angiography (OCTA) provides high-resolution, depth-resolving 3-dimensional visualization of the retinal microvasculature across all vascular layers. These characteristics make OCTA a potentially more sensitive imaging modality for detecting subtle microstructural remodeling.^34^, ^35^, ^36^, ^37^ Previous studies have also linked vessel geometry changes with both OCTA measurement and OCT-derived retinal geometry characteristics such as the retinal layer thickness.^35^^,^^38^^,^^39^ These findings suggest that integrating RVM features from fundal photographs with OCT and OCTA measurements could improve predictive performance. Future predictive models could combine these complementary imaging modalities to improve prognostic accuracy and clinical utility.^40^

Our study strengths include its large sample size and use of a fully automated, reproducible, open access RVM analysis deep learning tool (AutoMorph). The integration of AutoMorph allowed high-throughput extraction of RVM features from routine clinical images, minimizing observer bias. Furthermore, the use of macula-centered fundus images enhances clinical relevance, aligning with current imaging workflows for DR, and focusing on the region most critical for central vision. Nonetheless, several limitations merit consideration. First, the study was retrospective and hospital-based, and findings may not generalize to community-based DR screening populations. Second, data on potential confounding factors such as blood pressure, hemoglobin A1C, renal function, lipid profile, smoking status, and current medication were unavailable for adjustment and hence there is a risk of residual confounding. To partially mitigate this, we adjusted for baseline DR and DME severity grades, which strongly correlate with cumulative glycemic and systemic end-organ damage as a proxy of combined risk factor exposures. Third, as macula-centered rather than optic disc–centered fundus images were used, this may limit comparability with central retinal artery equivalent/central retinal vein equivalent findings from prior studies. However, the consistent association between macula RVM features with DR progression provides validating data to support the use of deep learning–based macula RVM analysis. Fourth, the study definition of incident treatment for STDR differs from the classical STDR definition as is does not include moderate to severe NPDR. Additionally, there may be changes in treatments and criteria for STDR over the study duration. However, while treatment criteria may change, the primary indications would relate to PDR and CSME. Fifth, we used images from a single device (Topcon 3-dimensional OCT) which may limit generalizability and multidevice validation is required. Finally, image quality may impact the segmentation of retinal vessels and subsequent RVM metrics, but AutoMorph excludes most low-quality images from analysis minimizing the overall impact on reported associations.

## Conclusions

Deep learning–derived macular RVM features autonomously extracted from standard macula-centered fundus images are significantly associated with incident treatment for STDR. Findings suggest that macular RVM features, in combination with other imaging features and clinical data, are potential biomarkers of DR progression that could be useful for prediction modelling. Future research should explore the utility of macular RVM features in prognostic models of incident STDR or its treatment and assess how RVM features change after STDR treatment to evaluate their role as biomarkers of treatment response. Future studies should also explore the role and utility of other multimodal retinal image biomarkers associated with DR progression that could complement RVMs such as capillary nonperfusion.

## Data Availability

Deidentified data from the MIDAS data set is not publicly available due to the absence of authorization for public data sharing. However, the data set is derived from a similar cohort that is available via INSIGHT with access/approval dependent on meeting data governance and application criteria.
