# Supplementary material for: Deep Learning–Derived Retinal Vascular Morphology and Incident Treatment for Sight-Threatening Diabetic Retinopathy
Source: Ophthalmol Sci. 2026 Aug 26;6(11):101385. doi: 10.1016/j.xops.2026.101385 (PMC13651561; doi:10.1016/j.xops.2026.101385)
Supplement: Supplementary file 1 [file mmc1.pdf]

Supplementary Material

Deep learning-derived retinal vascular morphology and incident treatment for sight-threatening diabetic retinopathy

Paul Nderitu[1,2,3*], Mark Chia[3,4], Urvi Karamchandani[1], Christine A. Kiire[5], Asterios Diafas[1], Sarah Matta[3,6], Yukun Zhou[2,3,7,8], Pearse A. Keane[1,2,3]

1. Moorfields Eye Hospital NHS Foundation Trust, London, United Kingdom
2. NIHR Biomedical Research Centre, Moorfields Eye Hospital NHS Foundation Trust, London, United Kingdom
3. Institute of Ophthalmology, University College London, London, United Kingdom
4. Department of Ophthalmology, Royal Victorian Eye & Ear Hospital, Canberra, Australia
5. Oxford Eye Hospital, Oxford University Hospitals NHS Foundation Trust, Oxford, United Kingdom
6. Université de Bretagne Occidentale, Brest, France
7. The UCL Hawkes Institute, University College London, London, United Kingdom
8. Department of Computer Science, University College London, United Kingdom

*Correspondence: p.nderitu@ucl.ac.uk

Supplementary Figure 1 - Baseline distributions of RVM features

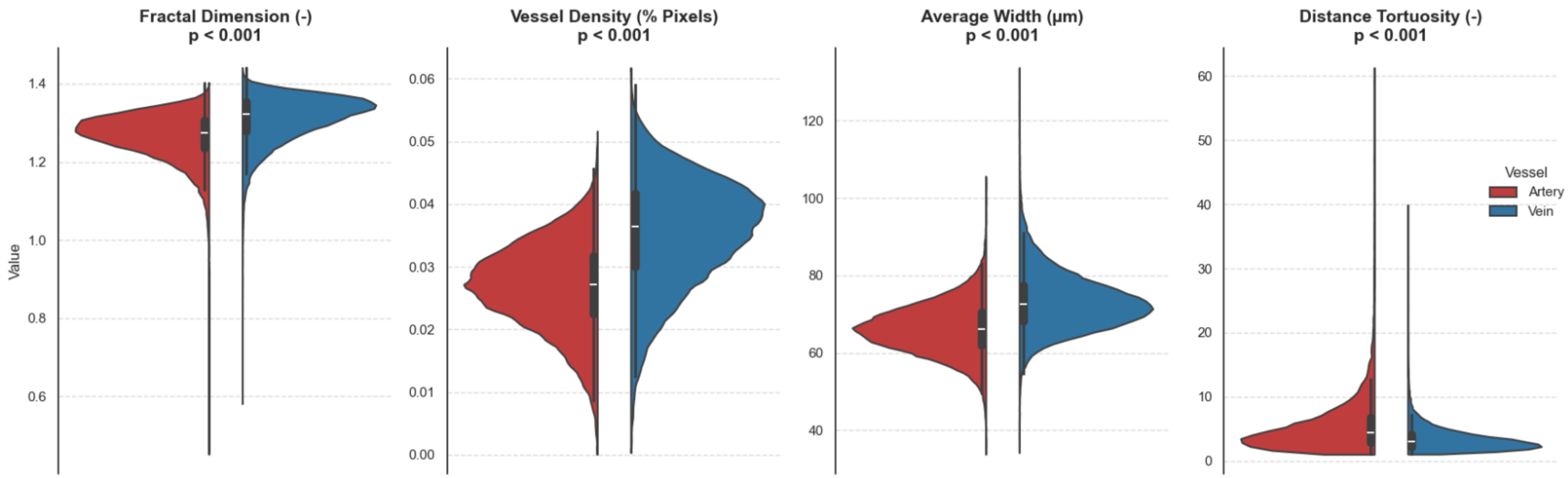

Violin plots show the distribution of four quantitative RVM features using AutoMorph comparing arteries and veins. *From left to right*: Vessel **complexity** (*fractal dimension – no units*), **density** (*vessel density % of pixels*), **caliber** (*average vessel width in* ***µm***), and **tortuosity** (*distance tortuosity – no units*). Arteries are shown in red and veins in blue. The width of each violin reflects the distribution density, with embedded boxplots indicating the median and interquartile range. Across the cohort, veins exhibit greater complexity, density, and caliber, while arteries demonstrate slightly higher tortuosity. **Significance test**: T-test with two-sided alpha of 0.05.

Supplementary Figure 2 – Correlation between RVM, age, DR grade and DME grade

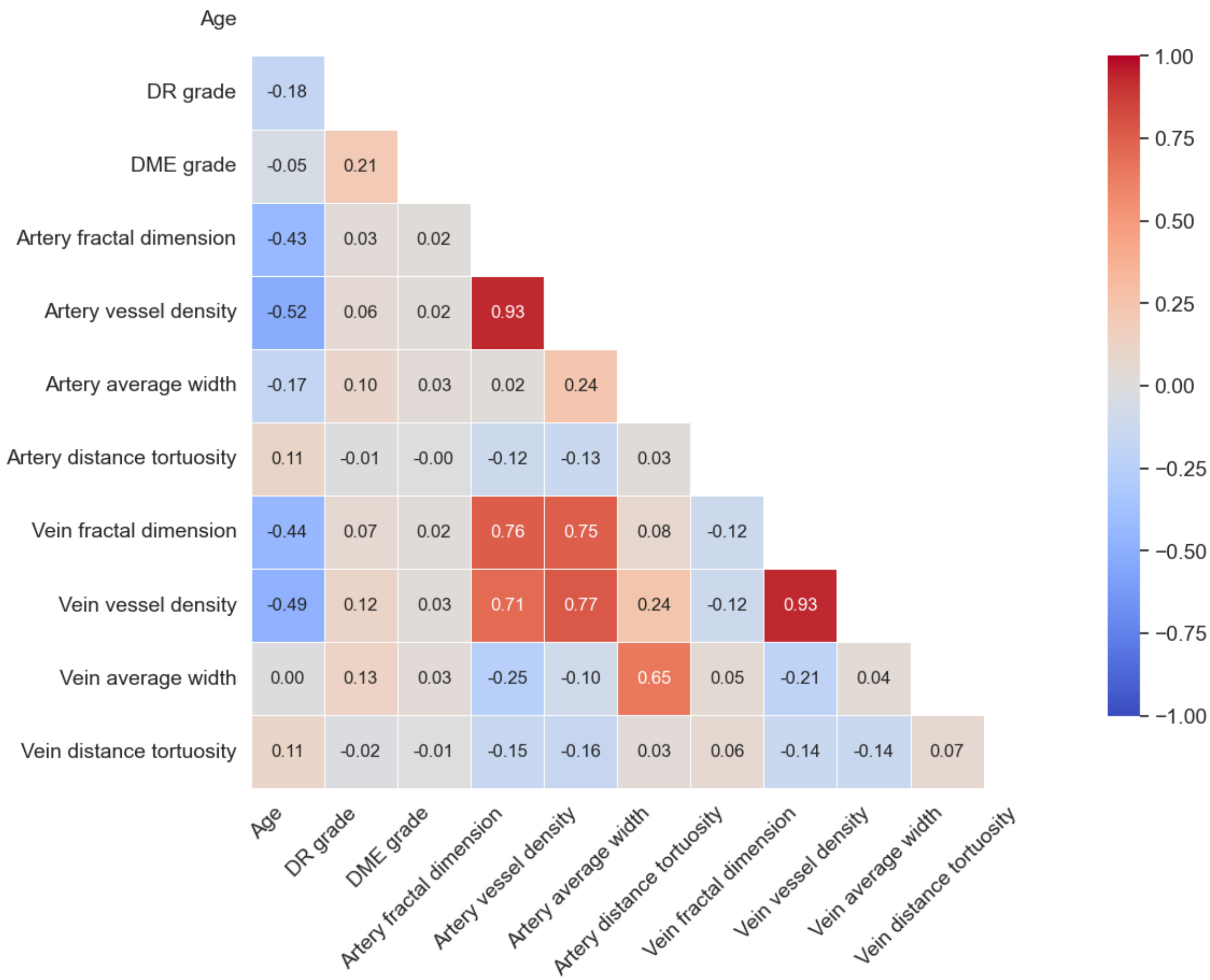

Pearsons's correlation coefficient matrix between various retinal vascular morphology (RVM) features, age, diabetic retinopathy (DR), and diabetic macula edema (DME) grade. Red hues indicate positive correlations, while blue hues indicate negative correlations, with color intensity corresponding to the strength of the correlation (-1.0 to 1.0). Artery and vein fractal dimension (complexity) shows a strong positive correlation with corresponding artery or vein vessel density. Artery and vein average width (caliber) are also correlated. Age demonstrates a moderate negative correlation with artery and vein fractal dimension and vessel density. DR grade exhibits a weak positive correlation with DME grade.

Supplementary Table 1 – Multivariate Cox regression in study cohort

RVM Feature	Outcome	Vessel	Q2[1] HR [95% CI]	Q3[1] HR [95% CI]	Q4[1] HR [95% CI]
**Complexity**	**STDR Treatment** *[3466 events]*	Artery	**0.8165*** [0.7398 – 0.9013]	**0.7072*** [0.6315 – 0.7920]	**0.5190*** [0.4516 – 0.5964]
		Vein	**0.8160*** [0.7399 – 0.9000]	**0.7180*** [0.6401 – 0.8054]	**0.6557*** [0.5717 – 0.7521]
	**PDR Treatment** *[1387 events]*	Artery	**0.7045*** [0.6002 – 0.8270]	**0.5986*** [0.4994 – 0.7174]	**0.4425*** [0.3562 – 0.5495]
		Vein	**0.7961*** [0.6785 – 0.9341]	**0.7229*** [0.6019 – 0.8684]	**0.6637*** [0.5354 – 0.8228]
	**CSME Treatment** *[2059 events]*	Artery	**0.8739*** [0.7711 – 0.9903]	**0.7692*** [0.6656 – 0.8888]	**0.5870*** [0.4898 – 0.7036]
		Vein	**0.8456*** [0.7469 – 0.9573]	**0.7119*** [0.6146 – 0.8246]	**0.6654*** [0.5564 – 0.7958]
**Density**	**STDR Treatment** *[3466 events]*	Artery	**0.7611*** [0.6889 – 0.8409]	**0.6151*** [0.5495 – 0.6887]	**0.4721*** [0.4116 – 0.5415]
		Vein	**0.9007*** [0.8141 – 0.9964]	0.9156 [0.8160 – 1.0273]	0.9100 [0.8160 – 1.0273]
	**PDR Treatment** *[1387 events]*	Artery	**0.6085*** [0.5175 – 0.7155]	**0.4770*** [0.3982 – 0.5713]	**0.3536*** [0.2857 – 0.4377]
		Vein	0.9506 [0.8064 – 1.1206]	0.9441 [0.7831 – 1.1283]	0.9441 [0.8111 – 1.2359]
	**CSME Treatment** *[2059 events]*	Artery	**0.8554*** [0.7536 – 0.9709]	**0.7192*** [0.6223– 0.8311]	**0.5811*** [0.4861 – 0.6947]
		Vein	**0.8751*** [0.7698 – 0.9948]	0.8981 [0.7759 – 1.0396]	0.8491 [0.7133 – 1.0107]
**Caliber**	**STDR Treatment**	Artery	**0.8534*** [0.7702 – 0.9531]	**0.8063*** [0.7210 – 0.9016]	**0.8020*** [0.7113 – 0.9043]

	[3466 events]	Vein	**1.2594*** [1.1217 – 1.4141]	**1.7455*** [1.5522 – 1.9628]	**2.5287*** [2.2346 – 2.8616]
	**PDR Treatment** *[1387 events]*	Artery	**0.7908*** [0.6671 – 0.9374]	**0.6509*** [0.5443 – 0.7785]	**0.6409*** [0.5303 – 0.7747]
		Vein	**1.2388*** [1.0215 – 1.5023]	**2.0477*** [1.6933 – 2.4763]	**3.2049*** [2.6262– 3.9110]
	**CSME Treatment** *[2059 events]*	Artery	0.9036 [0.7880 – 1.0361]	0.9326 [0.8050 – 1.0989]	0.9460 [0.8050 – 1.0989]
		Vein	**1.2541*** [1.0847 – 1.4498]	**1.5196*** [1.3089 – 1.7642]	**2.0504*** [1.7512 – 2.4008]
**Tortuosity**	**STDR Treatment** *[3466 events]*	Artery	**0.8477*** [0.7698 – 0.9335]	0.9148 [0.8317 – 1.0061]	1.046 [0.9485 – 1.1438]
		Vein	0.9900 [0.8994– 1.0898]	1.0290 [0.9357 – 1.1316]	**1.1137*** [1.0126 – 1.2247]
	**PDR Treatment** *[1387 events]*	Artery	**0.8416*** [0.7217 – 0.9815]	0.9036 [0.7762 – 1.0520]	**1.1945*** [1.0314 – 1.3833]
		Vein	0.9446 [0.8147 – 1.0951]	0.9047 [0.7793 – 1.0503]	1.0315 [0.8872 – 1.1991]
	**CSME Treatment** *[2059 events]*	Artery	**0.8726*** [0.7710 – 0.9876]	0.9303 [0.8234 – 1.0511]	0.9633 [0.8529 – 1.0879]
		Vein	0.9978 [0.8795 – 1.1321]	1.1000 [0.9723 – 1.2445]	**1.1624*** [1.0280 – 1.3143]

[1]Quartile 1 is the reference group. Multivariate Cox proportional hazards model adjusted for age, sex, ethnicity, baseline DR grade, baseline DME and a cluster term to adjust for inter eye correlation. Q: Quartile, HR: Hazard ratio, CI: Confidence interval, RVM: Retinal vascular morphology, PDR: Proliferative diabetic retinopathy, CSME: Clinically significant macula edema, Complexity: Fractal dimension, Density: Vessel density, Caliber: Average vessel width, Tortuosity: Distance tortuosity. Significant results (p<0.05) are indicated with a star (*) and are in **bold**.

Supplementary Table 2 – Multivariate Cox regression in patients with no/mild baseline DR

RVM Feature	Outcome	Vessel	Q2[1] HR [95% CI]	Q3[1] HR [95% CI]	Q4[1] HR [95% CI]
**Complexity**	**STDR Treatment** *[1417 events]*	Artery	**0.8011*** [0.6929 – 0.9263]	**0.7505*** [0.6296 – 0.8947]	**0.5111*** [0.4565 – 0.5723]
		Vein	**0.8107*** [0.7902 – 0.8316]	**0.7189*** [0.5807 – 0.8899]	**0.6905*** [0.6096 – 0.7821]
	**PDR Treatment** *[485 events]*	Artery	**0.6838*** [0.6372 – 0.7338]	**0.6080*** [0.4691 – 0.7880]	**0.3890*** [0.3658 – 0.4138]
		Vein	**0.8043*** [0.6859 – 0.9432]	**0.7644*** [0.6073 – 0.9620]	**0.7148*** [0.6871 – 0.7435]
	**CSME Treatment** *[932 events]*	Artery	0.8701 [0.7473 – 1.0130]	**0.8436*** [0.7597 – 0.9368]	**0.5938*** [0.5329 – 0.6616]
		Vein	**0.8343*** [0.8105 – 0.8588]	**0.7120*** [0.5817 – 0.8714]	**0.7126*** [0.5846 – 0.8685]
**Density**	**STDR Treatment** *[1417 events]*	Artery	**0.7478*** [0.6503 – 0.8599]	**0.5855*** [0.5707 – 0.6006]	**0.4506*** [0.4283 – 0.4740]
		Vein	0.9489 [0.7883 – 1.1421]	0.9605 [0.8914 – 1.0350]	1.0354 [0.9659– 1.1693]
	**PDR Treatment** *[485 events]*	Artery	**0.5742*** [0.5280 – 0.6244]	**0.4039*** [0.3689 – 0.4422]	**0.2774*** [0.2319 – 0.3320]
		Vein	0.9369 [0.6312 – 1.3908]	1.0282 [0.8951 – 1.1811]	**1.2875*** [1.0554 – 1.5706]
	**CSME Treatment** *[932 events]*	Artery	**0.8630*** [0.7541 – 0.9875]	**0.7222*** [0.7040 – 0.7480]	**0.5905*** [0.4861 – 0.6947]
		Vein	0.9608 [0.8813 – 1.0474]	**0.9479*** [0.9237 – 0.9727]	0.9470 [0.8743 – 1.0256]
		Artery	**0.8610***	**0.8475***	0.8513

Caliber	STDR Treatment [1417 events]		[0.7650 – 0.9691]	[0.7457 – 0.9631]	[0.6936 – 1.0450]
		Vein	**1.3245*** [1.0438 – 1.6806]	**1.8920*** [1.7652 – 2.0279]	**2.5975*** [2.4900 – 2.7096]
	PDR Treatment *[485 events]*	Artery	0.7712 [0.5381 – 1.1052]	**0.6310*** [0.5502 – 0.7237]	0.6139 [0.3543 – 1.0639]
		Vein	1.1318 [0.8217 – 1.5588]	**2.3660*** [2.0720– 2.7020]	**3.7195*** [3.0146 – 4.5893]
	CSME Treatment *[932 events]*	Artery	**0.9299*** [0.9145 – 0.9456]	0.9902 [0.8862 – 1.1064]	1.0101 [0.9991 – 1.0211]
		Vein	**1.3641*** [1.1058 – 1.6826]	**1.6287*** [1.3637 – 1.9451]	**2.0668*** [1.7250 – 2.4763]
Tortuosity	STDR Treatment *[1417 events]*	Artery	**0.8532*** [0.8222 – 0.8855]	**0.9030*** [0.8383 – 0.9726]	0.9820 [0.7723 – 1.2589]
		Vein	1.0102 [0.9931– 1.0276]	**1.0867*** [1.0021 – 1.1316]	1.1582 [0.8995 – 1.4912]
	PDR Treatment *[485 events]*	Artery	**0.8401*** [0.7215 – 0.9783]	0.8481 [0.6354 – 1.1152]	1.1386 [0.8760 – 1.4800]
		Vein	0.9722 [0.8467 – 1.1162]	0.9486 [0.7793 – 1.0503]	1.1306 [0.8676 – 1.4733]
	CSME Treatment *[932 events]*	Artery	**0.8557*** [0.7577 – 0.9665]	0.9274 [0.7314 – 1.1758]	0.9260 [0.7125 – 1.2036]
		Vein	1.0151 [0.9257 – 1.1132]	1.1550 [0.9896 – 1.3480]	1.1678 [0.9113 – 1.4966]

[1]Quartile 1 is the reference group. Multivariate Cox proportional hazards model adjusted for age, sex, ethnicity, baseline DR grade (mild) and a cluster term to adjust for inter eye correlation. Q: Quartile, HR: Hazard ratio, CI: Confidence interval, RVM: Retinal vascular morphology, PDR: Proliferative diabetic retinopathy, CSME: Clinically significant macula edema, Complexity: Fractal dimension, Density: Vessel density, Caliber: Average vessel width, Tortuosity: Distance tortuosity. Significant results (p<0.05) are indicated with a star (*) and are in **bold**.

Supplementary Figure 3 – Macula images from low and high RVM quartiles

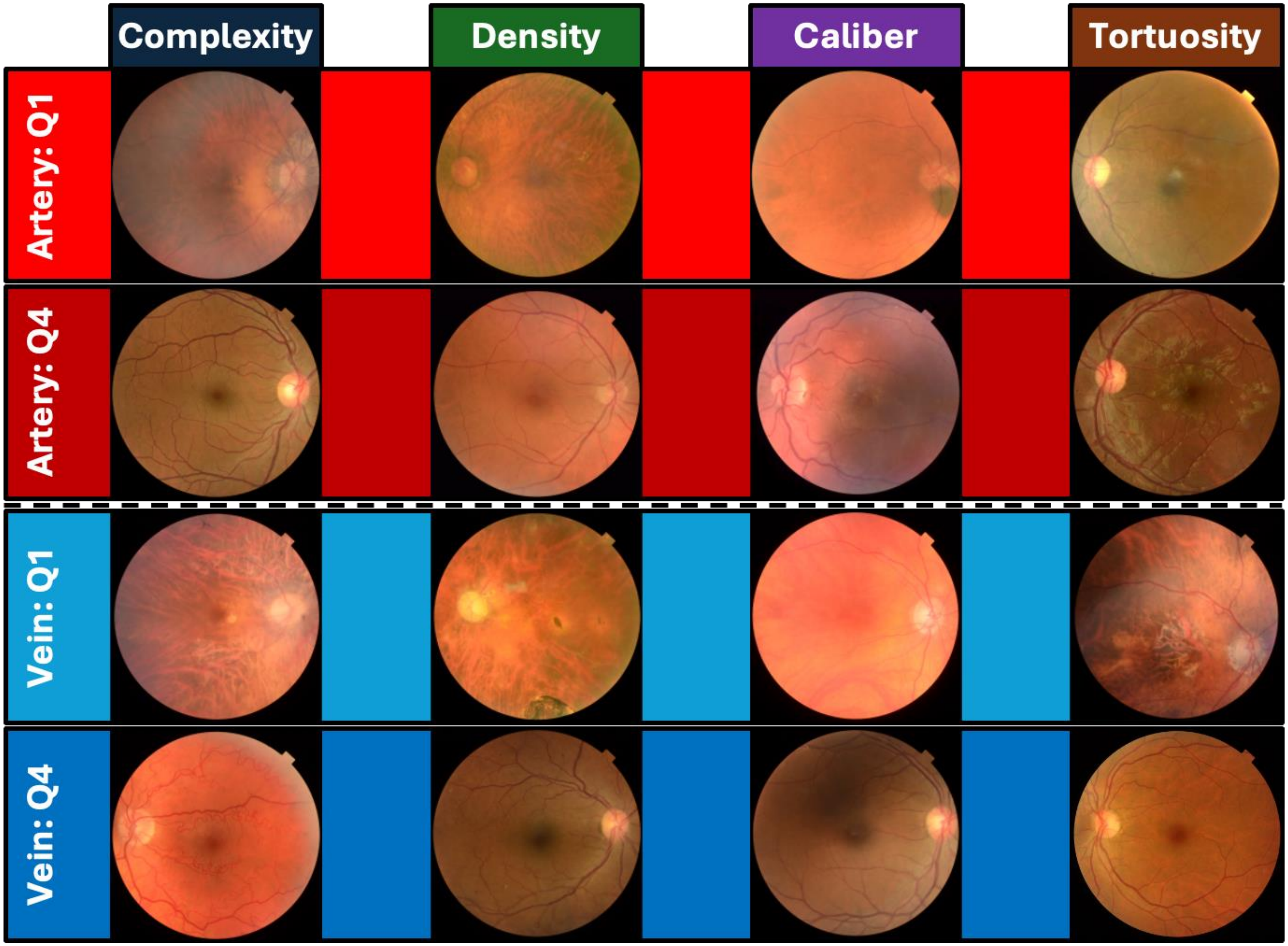

A sample of macula images from the lowest (Q1) and highest (Q4) RVM feature quartile for arteries and veins. Images show that RVM features align with clinically plausible appearances of the RVM features in eyes from lower and higher quartile groups. Q: Quartile, RVM: Retinal vascular morphology.
